# Malignant pleural effusion: current understanding and therapeutic approach

**DOI:** 10.1186/s12931-024-02684-7

**Published:** 2024-01-19

**Authors:** Francesca Gonnelli, Wafa Hassan, Martina Bonifazi, Valentina Pinelli, Eihab O Bedawi, José M. Porcel, Najib M Rahman, Federico Mei

**Affiliations:** 1 Department of Biomedical Sciences and Public Health, Università Politecnica delle Marche, Ancona Via Conca 71, Ancona, 60126 Italy; 2 Department of Respiratory Medicine, Sheffield Teaching Hospitals, University of Sheffield, Sheffield, UK; 3 Pulmonology Unit, San Bartolomeo Hospital, Sarzana, Italy; 4 Research Group of Cancer Biomarkers, Lleida Institute for Biomedical Research Dr. Pifarré Foundation (IRBLleida), Lleida, Spain; 5 Pleural Medicine and Clinical Ultrasound Unit, Department of Internal Medicine, Arnau de Vilanova, University Hospital, Lleida, Spain; 6 Oxford Pleural Unit, Oxford University Hospitals NHS Foundation Trust, Churchill Hospital, Oxford, UK; 7 Oxford NIHR Biomedical Research Unit, Oxford, UK; 8 Chinese Academy of Medicine Oxford Institute, Oxford, UK

**Keywords:** Malignant pleural effusion (MPE), Pleural, Oncology, Thoracoscopy, Pleurodesis, Chest drain, Indwelling pleural catheter (IPC)

## Abstract

Malignant pleural effusion (MPE) is a common complication of thoracic and extrathoracic malignancies and is associated with high mortality and elevated costs to healthcare systems. Over the last decades the understanding of pathophysiology mechanisms, diagnostic techniques and optimal treatment intervention in MPE have been greatly advanced by recent high-quality research, leading to an ever less invasive diagnostic approach and more personalized management. Despite a number of management options, including talc pleurodesis, indwelling pleural catheters and combinations of the two, treatment for MPE remains symptom directed and centered around drainage strategy. In the next future, because of a better understanding of underlying tumor biology together with more sensitive molecular diagnostic techniques, it is likely that combined diagnostic and therapeutic procedures allowing near total outpatient management of MPE will become popular. This article provides a review of the current advances, new discoveries and future directions in the pathophysiology, diagnosis and management of MPE.

## Introduction

Malignant pleural effusion (MPE) is the accumulation of fluid between the lung and the chest wall as a result of cancer cells in the pleura. MPE is a common complication of cancer, with an estimated incidence of 500,000 new cases in the USA and Europe combined [1, 2]. MPE can occur in up to 20% of people with cancer and can be associated with any type of cancer, both primary pleural malignancy (mesothelioma) and the result of secondary spread from other sites including lung, breast, and ovarian neoplasm [3]. It is estimated that the global incidence is 70 per 100,000 [4] and in the United States it accounted for 361,270 hospital admission costing $10.1 billion in 2016 [5]. Therefore, the impact of MPE on healthcare system is not negligible, being cost-consuming due to the high rate of hospital readmission and resources use [5].

The presence of MPE is associated with poor patient quality of life, due to breathlessness, pain, cachexia, fatigue and reduced daily activity and a bad prognosis with a median survival rate of 3–12 months [6, 7] Patient prognosis is extremely variable, depending on several factors such as primary cancer type, stage, and performance status. Regarding the latter, to date, two prognostic scoring system have been validated in MPE: the LENT and PROMISE scores [8, 9]. Both systems adopt a combination of clinical and biological variables, including tumor type and laboratory tests, but, despite their simplicity and external validation, there has been a suggestion that further detailed scores are needed [10], and these scores may not hold validity in light of new targeted therapy for lung cancer and molecular subtypes. Although evidence base for the diagnosis and management of MPE has significantly improved in the last decade, current treatment remains palliative, aiming for symptom relief by multiple approaches to fluid management, including repeated aspiration, chest tube and talc slurry, talc poudrage at medical thoracoscopy (MT) and Indwelling Pleural Catheter (IPC) with or without talc slurry. Therefore, pleural fluid removal is currently recommended only in symptomatic MPE patients [4]. Besides meaningful clinical progress on diagnosis and treatment of MPE, over the last decade significant advances have also been achieved in the pathophysiology of MPE and the biological properties of pleural fluid [11, 12].

Hence, this article aims to review the recent advances in pre-clinical research and standards of care in the management of patients suffering with MPE.

## Pathogenesis of malignant pleural effusion

### Pleural invasion of malignancy

Although the pleura may be invaded through lymphangitic spread or by the infiltration from adjacent structures (i.e., diaphragm, pericardium, chest wall), data from autopsy studies showed that tumor cells spread to the pleura mainly by the bloodstream, initially invading the visceral pleura. Therefore, secondary diffusion to the parietal pleural occurs by tumor seeding along adhesions or by exfoliated malignant cells floating in the fluid [13]. Once arrived at the parietal pleura, tumor cells adhere to the mesothelium, evading pleural immune defense mechanisms, invading pleural tissue, and gaining access to nutrients and growth factors. In MPE patients, a complex interaction between tumor and host cells results in a pleural immunosuppressive environment, mainly due to impaired macrophages and lymphocyte cytotoxic function as well as a massive production of pro-inflammatory and tumor-stimulating mediators [14]. Despite their detachment from pleural tissue, tumor cells floating in pleural space are still able to form secondary foci in other sites of the cavity, which suggests their capacity to use alternative sources of energy and growth factors. Recent data from early translational work showed that cancer cell cultures proliferation is promoted by seeding the cells in pleural effusion regardless of the source, suggesting a pro-growth property of pleural fluid [15]. Therefore, it is arguable that pleural fluid may not be a bystander of malignancy, but may be an active promoter of cancer progression, thus leading to a potential change in MPE treatment approach, no longer symptoms relief-focused but aiming to achieve an early pleural fluid control.

### Malignant pleural effusion production mechanisms

Pleural fluid accumulates when production is greater than drainage. The reason why some tumors cause effusions while others do not is unclear. Absorption is reduced when tumors invade the drainage system, anywhere from parietal pleural stomata to hilar and mediastinal lymph nodes [16]. However, blockade of fluid removal alone is not adequate to explain MPE formation as the following aspects seem to demonstrate: (1) in most MPE patients there is a dissociation between pleural fluid volume and tumor extent; (2) the protein content is higher in malignant fluids than in normal pleural fluids, suggesting the presence of plasma leakage; (3) MPE occurs even in patients without parietal pleura involvement [17–19]. It is therefore currently believed that a combination of increased fluid production due to fluid extravasation from hyper-permeable parietal or visceral pleural and/or tumor vessels and impaired lymphatic outflow underlie the development of MPE.

The interaction between tumor and host cells, including mesothelial, endothelial, myeloid, and lymphoid cells, contributes to the release of vasoactive mediators. The balance between permeability-stimulating (e.g., vascular endothelial growth factor – VEGF, tumor necrosis factor – TNF, osteopontin – OPN, etc.) and inhibitors (e.g., endostatin) molecules plays a crucial role in MPE development [14].

### Cell and molecular biology in malignant pleural effusion

It is known that MPE is a protein-rich fluid including growth factors and cytokines with pro-inflammatory, oncogenic and angiogenic properties such as VEGF, and immunosuppressive molecules such as interleukin-10 (IL-10) [20]. This suggests the hypothesis that MPE provides a nutrient-rich microenvironment to support tumor growth, while suppressing anti-tumor immune activity.

**Fig. 1:**
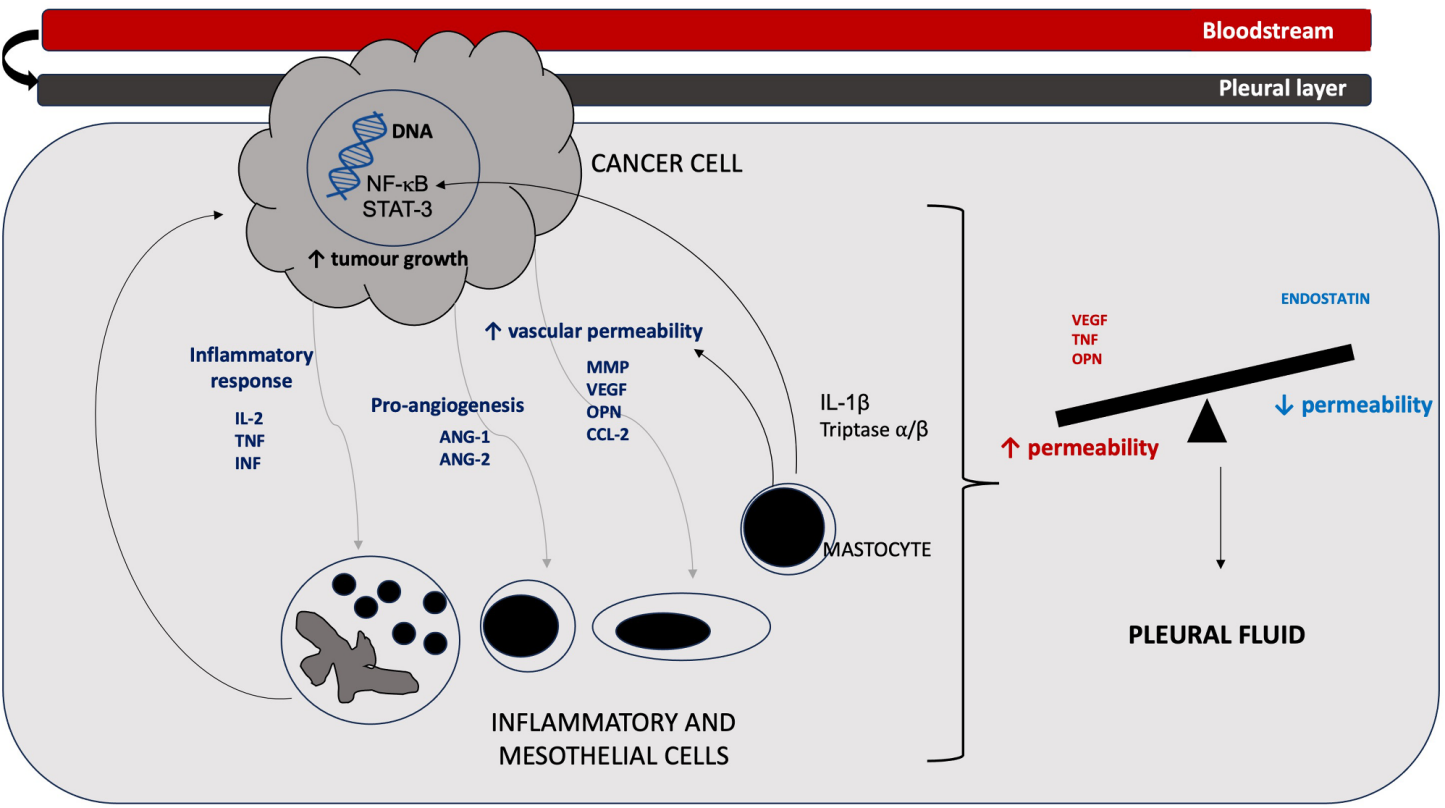
MPE pathogenetic mechanism

The tumor-host cells interaction underlies these effects in pleural space changes by means of a wide production of molecules, which can be divided, according to their proprieties, in three categories: (1) factors stimulating pleural inflammation (e.g., interleukin 2 - IL2, interleukin 6 – IL6 and TNF); (2) pro-angiogenesis mediators (e.g., angiopoietin 1 and 2 – (ANG-1 and 2); (3) particles promoting vascular hyperpermeability (e.g., VEGF, matrix metalloproteinases—MMP, chemokine (c-c motif) ligand 2—CCL, OPN, etc.) [21]. Finally, evidence from in vivo studies showed that these pathogenetic events are triggered by tumor cells, executing pro-inflammatory and pro-angiogenetic transcriptional programs (transcription factors nuclear factor – (NF)-κB; signal transducer and activator of transcription – STAT 3) [22–25]. A possible role in MPE production can be played by mastocytes releasing of tryptase alpha/beta 1 and interleukin-1β (IL–1β), which increase pulmonary vessels permeability and promote fluid accrual and tumor growth production by (NF)-κB activation (Fig. 1) [26]. Concerning genetic mutations, data from genomic studies reported that activation of *EGFR*, *KRAS*, *PIK3CA*, *BRAF*, *MET*, *EML4*/*ALK* and *RET* mutations are related to increasing MPE formation, being, particularly, *KRAS* mutations most common in distant metastases and *EGFR* mutations in tumors with regional metastatic infiltration [27].

## Diagnosis

Together with patient symptoms, early radiological detection of a predominantly unilateral pleural effusion represents another fundamental step when MPE is suspected. In this context, the chest radiograph remains useful, being the most rapidly available diagnostic tool. However, it is known that no single test can provide the complete answer, but a combination of a clinical assessment, evaluation of pre-test probabilities, and a careful selection of diagnostic tests allows physicians to be more confident in their diagnosis. Over the last decade significant advances have been achieved in diagnosing of pleural disease, particularly due to the use of thoracic ultrasound (TUS), which now plays a crucial role in diagnostic work-up of pleural conditions, including MPE. Since the initial pleural aspiration may have limited utility in the diagnosis of MPE, due to its low sensitivity, histological analysis of pleural tissue obtained via biopsy is usually required to get a definitive diagnosis and guide oncological treatment, although not in all cases where sometimes fluid alone is sufficient for both diagnosis and molecular testing of malignancy. However, data suggests this occurs in the minority of cases (around 30% in one analysis) [28].

### Non-invasive

#### Ultrasonography

Thoracic ultrasound has revolutionised the diagnosis and delivery of care in pleural disease including MPE.

Qureshi et al. [29] reported a sensitivity of 73% and a specificity of 100% of TUS in distinguishing between malignant and benign pleural diseases, being pleural thickening exceeding 1 cm, pleural nodularity, and diaphragmatic thickening greater than 7 mm suggestive of malignancy.

Notably, discrete metastatic pleural nodules (greater than 5 mm) are readily detectable on TUS when MPE is present, due to the excellent acoustic window created by the anechoic appearance of the pleural fluid. Additionally, TUS can be useful and accurate also to detect chest wall involvement in patients with lung cancer and suspected tumour growth beyond parietal pleura [30].

Although TUS does not allow to achieve a definite MPE diagnosis, it can show suggestive signs requiring further invasive investigations. Furthermore, TUS is currently recommended to guide most of pleural interventions procedures minimizing the risk of adverse events, such as accidental organ puncture, pneumothorax, and bleeding from intercostal and internal mammary arteries [31–34].

Moreover, a pre-thoracoscopy TUS assessment can aid the operator in selecting the optimal entry site, allow for the recognition of the sliding sign as an indicator of lung collapsibility and predict non-expandible lung [35, 36].

#### CT-scan

Contrast-enhanced chest computed tomography (CT) remains an essential step in diagnosing MPE, providing pleural features indicative for malignancy such as nodular pleural thickening, mediastinal pleural thickening, parietal pleural thickening (> 1 cm) and circumferential pleural thickening, showing a high specificity but low sensitivity [37, 38].

Porcel et al. designed a single-centre study to validate a scoring system for identifying malignancy based on chest CT findings that included three elements: (1) Pleural lesion greater than or equal to 1 cm (5 points); (2) Presence of liver metastasis, abdominal mass, or lung mass/nodule (3 points each); (3) Absence of pleural loculations, pericardial effusion, or cardiomegaly (2 points each) A total score of 7 points or higher predicted MPE with a sensitivity of 88% and a specificity of 94% in the validation cohort [39].

Whether or not pleural fluid drainage before CT scan is required, is debatable, as scanning prior to pleural tap allows a better visualisation of the pleural layer but doesn’t provide accurate information on potential lung parenchymal lesions. In addition, the presence of pleural fluid may facilitate additional interventional procedures, such as thoracoscopy [40]. Nevertheless, there is no evidence of a clear advantage of either of the two approaches, as demonstrated by Corcoran et al. [41]. Therefore, the choice of further invasive procedure to obtain a pleural biopsy should be guided by the clinical scenario and local resources.

#### FDG-PET scan

18 F-fluorodeoxyglucose – positron emission tomography (PET-CT) is a functional imaging technique which contemporarily provides anatomical CT reconstructions and metabolic behaviour of the examined district. Generally, malignant cells present an increased uptake of 18 F-fluorodeoxyglucose, whereas benign tissue are less metabolically active.

In contrast to the setting of mesothelioma staging [6], where PET-CT plays a crucial role, studies regarding the potential diagnostic contribution of PET-CT in MPE have provided conflicting results. The main limitation of PET-CT use in MPE is potential false-positive diagnosis in case of pleural inflammation/infection or after talc pleurodesis [42, 43].

Several meta-analyses have been conducted aiming to provide a pooled estimation of diagnostic accuracy of PET-CT in the differentiation of malignant and benign pleural conditions, but the results are still not conclusive. Data from a meta-analysis by Porcel et al., focused on qualitative/visual readings and semi-quantitative readings, revealed that qualitative/visual methods yielded a high sensitivity of 91%, but a low specificity of 67%, leading to a higher likelihood of false-positive results [44].

Finally, a more recent metanalysis by Fijaellegaard et al. focused specifically on MPE, showed that visual/qualitative image analysis was superior to semi-quantitative in detecting pleural malignancy [45]. Thus, PET-CT scanning in the workup of MPE is not routinely recommended, but it might be useful in providing specific targets for image-guided pleural biopsies where medical thoracoscopy is precluded or have failed to guarantee a definite tissue diagnosis [46].

### Invasive

#### Thoracentesis

Thoracentesis with pleural fluid analysis is usually the first invasive approach in the suspicion of MPE, that commonly exhibits exudative features with a net predominance of mononuclear cells [47].

Even though pleural fluid cytology is the essential part of pleural fluid analysis, both sensitivity and specificity are suboptimal and vary depending on the primary cancer type.

Porcel et al. carried out a large retrospective study reporting an overall sensitivity of 51% [48]. Likewise, overall sensitivity was confirmed in a prospective cohort study, which reported a value of 46% and showed that the diagnostic accuracy of pleural fluid analysis depends on cancer histotype, ranging from a significantly low accuracy in mesothelioma (6%) and hematological malignancies (40%) to higher value in adenocarcinomas (79%) (up to 95% in ovarian adenocarcinomas) [49].

Interestingly, Mercer et al. found a negative association between pleural thickening on CT and both negative cytology (*p* < 0.001) and insufficient samples (*p* = 0.001) [28].

Several attempts to improve the diagnostic accuracy of pleural fluid cytology have been made. Literature data report that while a larger amount of pleural fluid volume doesn’t impact on sensitivity, a second, but not subsequent, pleural aspirations might be useful in increasing the diagnostic yield [48, 50, 51].

However, to optimize the utility of the cell block preparation, it might be suggested to submit at least 50 ml of pleural fluid.

Furthermore, additional immunocytochemistry tests, such as BAP1 loss and p16 deletion may help distinguishing between benign pleural effusion and MPE, particularly benign mesothelial hyperplasia from malignant pleural mesothelioma [52–54].

#### Image-guided pleural biopsies

Pleural biopsy is still the essential diagnostic step for MPE, particularly when pleural fluid analysis is inconclusive.

The most commonly used pleural biopsy techniques include ultrasound-guided or CT-guided pleural biopsy using a cutting needle visualised under image guidance.

CT-guided pleural biopsy has an excellent diagnostic yield providing adequate tissue for diagnosis in almost 90% of cases [55], but also shows several limitations, such as the use of ionizing radiation and the reliance on breath-holding techniques.

TUS-guided pleural biopsy is typically faster to undertake, can be conducted by physicians, does not expose patients to ionizing radiation and can be combined easily with therapeutic drainage procedures such as chest drain or IPC insertion. A key caveat that clinicians must bear in mind in regard to image guided biopsy techniques, is that the diagnostic yield is likely to be significantly dependent on the presence of an adequate ‘target’ identifiable with the imaging technique.

A systematic review and metanalysis by Mei et al. comparing the diagnostic yield of TUS and CT-guided pleural biopsy revealed excellent pooled results for both these procedures, being respectively 84% and 93% [56]. It’s noteworthy that the number of studies reporting US-guided biopsy was three-fold higher than that of studies on CT, suggesting a widespread adoption and use of this technique in clinical practice [56].

Consistent results were also found in a retrospective study by Mychajlowycz et al., who reported an equivalent sample adequacy for both modalities (88% for US and 92% for CT, *p* = 0.53) [57]. Even better values were reported in a large retrospective study, comprising both pleural-based lesions as well as peripheral lung lesions, and a diagnostic success was achieved in 100 of 103 TUS-guided procedures (97.1%) and in 164 of 170 CT-guided procedures (96.5%) (*p* = 0.999) [58].

#### Medical thoracoscopy

Despite the growing body of evidence supporting less invasive procedures like TUS- and CT-guided pleural biopsy, medical thoracoscopy (MT) remains the gold standard for diagnosis of MPE. It allows the operator to explore the contents of the pleural cavity, guide biopsy to the most impaired areas under direct vision, remove pleural fluid and to perform pleurodesis.

MT can be performed by trained interventional pulmonologists in endoscopy suite under local anesthesia and conscious sedation. The direct sampling of pathological areas visible on the pleural layer leads to an excellent sensitivity and specificity [59, 60]. In this context, several studies have reported accuracy exceeding 90% [59, 60], with values close to 100% for MPM [61].

Absolute contraindications to MT include (1) lung adherent to the chest wall throughout the hemithorax; (2) respiratory failure precluding safe sedation; (3) uncontrollable cough [3].

The traditional instrument is the rigid thoracoscope, but more recently, a semiflexible device has been increasingly adopted. It offers the advantage of an increased manoeuvrability, similarities in its functional design to the flexible bronchoscopy and the compatibility of the same equipment. On the other hand, its smaller working channel may limit size and depth of biopsies [47]. However, these technical differences between rigid and semi-rigid thoracoscope do not appear to significantly impact the MT diagnostic performance [62]. Dhooria et al. reported a better absolute overall sensitivity when MT was performed with rigid instruments on an Intention to Treat (ITT) analysis, but the yield was similar comparing only those in whom ultimate biopsy was achieved [62].

Attractive tools, recently proposed to overcome difficulties in sampling fibrous pleura and to gain larger biopsies via semirigid thoracoscopy, are insulated-tip diathermic knife and cryoprobe [47]. Technical feasibility, safety and diagnostic yield of pleural sampling using the flexible cryoprobe has been investigated in several studies [63, 64], summarized in two recent meta-analyses showing that, compared with flexible forceps, pleural cryobiopsy obtained larger and deeper tissue specimens with less crush artifacts but does not show superiority for diagnostic yield [65, 66]. However, these techniques (rigid, semiflexible, cryobiopsy MT) should not be necessarily intended as competitive, as ideally, in interventional pulmonology centers all above options should be available to tailor the procedure according to patients’ characteristics.

Although literature data suggested a possible step-by-step approach, starting with less invasive techniques and progressing to more invasive ones [67], the optimal diagnostic work-up in suspected MPE is still to be established. MT remains the gold standard due to diagnostic and therapeutic meaning and, thus, it should be pursued as a first line approach, particularly where malignant mesothelioma is supposed [4].

## Update in management of malignant pleural effusion

Over the course of the years research has made significant progress in defining the optimal management of MPEs, a question which remains an important topic of ongoing research given the burden on patients, their careers and healthcare systems.

A decade ago, there was little supporting research to inform recommendations [68] which were largely linear and non-personalised. In the last decade there have been a number of randomised controlled trials (RCTs) addressing drain size [69], comparing talc administration methods [70], optimal methods of achieving pleurodesis [71, 72] IPC drainage strategies [73, 74] as well as combination approaches [75, 76].

Thus the recently updated British Thoracic Society (BTS) pleural guidelines in 2023 [4] have outlined a variety of procedures and strategies which can be used to alleviate symptoms of dyspnoea caused by MPEs, centring not only around prognosis and lung re-expandability but also patient priorities and preference of an inpatient versus ambulatory strategy, accepting there is no ‘right’ definitive management option. However, whilst there has been considerable progress in trials focusing on patient-centred outcomes such as improvement in dyspnoea scores and quality of life measures, further work is needed to explore what the psychosocial aspects of living with MPE, its intendent interventions and how as a community of clinicians caring for these patients, we best support them.

### Pleurodesis

Assessing lung re-expandability and excluding ‘trapped lung’ remains the initial step in directing the treatment pathway. Whilst more novel methods using ultrasound techniques such as M-mode are being studied to predict non-expansile lung (NEL), in clinical practice this is most commonly determined by an initial large volume therapeutic aspiration (usually up to 1.5 L or earlier if symptoms such as cough or chest pain occur) followed by a chest radiograph. This has the additional benefit of ensuring symptomatic benefit from pleural fluid drainage as if the patient is unlikely to benefit, a conservative ‘watchful waiting’ strategy may be more appropriate. In patients with an expected prognosis of less than 30 days, recurrent aspiration remains appropriate, and a more definitive strategy is not expected to confer any additional patient benefit.

Achieving a pleurodesis has been and continues to be an important goal in the management of MPE. A ‘successful’ pleurodesis is one that prevents the patient from needing a further therapeutic intervention for their MPE, usually measured at 90 days [70]. A recent network meta-analysis of 80 randomised studies (*n* = 5507) identified talc as the most ‘efficacious agent’ in inducing pleurodesis (via slurry or poudrage) compared to other agents such as bleomycin and doxycycline, IPC and placebo [77]. Historically, chest drain insertion followed by talc slurry or thoracoscopically applied talc ‘poudrage’ have been the most common pleurodesis modalities.

With regards to chest tube size, we know from the TIME 1 study [69] that a larger size (18–24 F) may confer an additional benefit compared to 12 F primarily thought to be due to reduce displacement and tube blockage. Pleurodesis is painful and the TIME-1 study provided evidence that there is no contraindication to the use of non-steroidal anti-inflammatory medications as an option for analgesia and their use during pleurodesis was not associated with lower success rates [69].

The landmark study by Dresler et al. [78] demonstrated no differences between pleurodesis success at 30 days comparing talc slurry versus poudrage. However, in recent years the study result has been widely debated largely due to critique of its trial design using a radiological outcome and early assessment of the primary endpoint at 30 days. The TAPPS study conducted by Bhatnagar et al. [70] addressed this specifically using a more modern trial design with talc poudrage using the now commonplace medical (local anaesthetic) thoracoscopy (LAT) compared with talc slurry through a 12-14 F chest tube. TAPPS used a clinical definition of pleurodesis (as described above) as its primary endpoint assessed at a longer interval at 90 days. No difference was reported between the groups in failure rates, although, as the authors recognized themselves, the study may have been underpowered to detect small but potentially important differences [70].

Surgical options such as video-assisted thoracoscopic surgery partial pleurectomy (VATS-PP) was compared to ‘physician performed’ talc pleurodesis in the MesoVATS study (*n* = 175 mesothelioma patients). VATS-PP was associated with longer hospital stay, was more expensive and associated with more complications without any difference in fluid control or quality of life [79]. The recently updated BTS guidelines highlight that there is insufficient evidence as to whether a surgical pleurodesis or decortication is better than talc slurry pleurodesis and suggest that, in selected patients considered fit enough for both modalities and where accessibility is not a barrier, both techniques should be discussed to individualise treatment choice.

The limitations of chest drain slurry or talc poudrage via LAT remain the duration of hospital stay for these patients. As stated previously the median survival in literature of these patients varies from 3 to 12 months [6] and studies quote a mean survival of 4 months [71], although this is likely longer now with developments in anticancer therapies. The median stay has now been quoted as 2–3 days (the use of ultrasound has shown to shorten the stay by 1) [80], the pain associated with administration of talc and the failure rate of pleurodesis [70], these factors combined may for some patient cohorts lead them to seek more of an ambulatory approach for the management of their MPEs [4].

### Indwelling pleural catheters

IPCs provide the option of managing MPE in the home setting and insertion is done as a day case procedure [6]. Compared to chest tube and talc pleurodesis, the question to ask is which is better when it comes to improving dyspnoea scores and quality of life. The TIME 2 trial looked at both these entities the primary outcome being the difference in the degree of dyspnoea at 42 days between the two groups. The outcome was both these modalities were as effective as the other in terms of improving dyspnoea and neither offered any significant benefit over the other in regard to quality of life or breathlessness [71]. The outcome of this study is reflective in the current BTS guidelines which suggest that either of these should be offered as a first line of management to patients with MPEs [4].

With shorter hospital stays, cost effectiveness in the initial 14 weeks period, and the reduced need for further pleural procedures [72] as well as the occurrence of auto pleurodesis in upto 37.2% of patients [74] one could argue that IPCs should be given preference over chest drain and talc slurry [71]. However, it is important to remember that IPCs are associated with their own set of adverse events which include infection quoted at 5% [81], catheter blockage and site cellulitis to name a few [71]. In a retrospective study of the patient journey with an IPC until removal or death (*n* = 181), patients required a median of 96 individual home drainages with potential not negligible loss of protein. Approximately one third of patients required at least one review in hospital and 23% more than one review. Thus, the true burden of IPC-related treatment is likely underestimated given the sparsity of qualitative data offering a patient perspective and sufficient data reflecting the related health economics [77]. Additionally, with an IPC serving as a constant reminder of a terminal cancer, what also remains understudied are the psychosocial aspects of IPCs [82]. Whilst on one hand, IPC may empower patients to have more control of their disease, limitations on functional and leisure activities are often understated [83]. Simple tasks such as taking a shower, getting dressed and even sleeping can be troublesome and anxiety-provoking for patients, some who frequently worry about damaging their IPC [83].

Studies have also investigated what influences rates of auto pleurodesis as this would lead to earlier removal and avoidance of the adverse effects associated with IPCs. Frequency of fluid drainage was an aspect explored by the ASAP and AMPLE-2 studies and whether aggressive drainage would facilitate increased rates of pleurodesis enabling earlier IPC removal. Both the ASAP and AMPLE-2 trials were able to show that aggressive daily drainage of pleural fluid is associated with higher rates of auto pleurodesis [73] and IPC removal [72]. The pathophysiology behind this is related to the idea that an empty pleural cavity allows for better apposition of the visceral and parietal pleural surfaces and thus adhesion of the two surfaces together [84]. Of note increased frequency of drainage did not influence breathlessness scores when compared to the symptom-guided drainage group. However, in AMPLE-2 aggressive drainage was potentially associated with improved quality of life (QOL measures). No adverse features such an increase in the incidence of pleural infection or pain associated with daily drainages was seen in the aggressive drainage group [74].

The SWIFT trial was a single-blinded multicentre RCT evaluating whether a novel silver nitrate-eluting indwelling pleural catheter (SNCIPC) [85] could improve pleurodesis efficacy at 30 days compared to a standard indwelling pleural catheter. Although no significant difference in treatment-related adverse events was observed, the study was negative and did not support wider use or evaluation of the SNCIPC device.

### Combination approaches

It is clear from the above that there are different merits and advantages to the varied modalities. While IPC provides an ambulatory management option, it is a far inferior pleurodesis agent to talc. This led to the recent interest in combination approaches. The IPC-PLUS trial was a single blinded multi-centre study [75], where patients with symptomatic MPE underwent regular daily drainage for 10 days and provided there was no significant evidence of NEL, they were randomised to intrapleural instillation of talc slurry versus normal saline (placebo) with patients blind to treatment. The primary outcome was pleurodesis success rate at five weeks, and this was almost doubled in the intervention arm (43% vs. 23%). What is interesting to note is that despite this being an enriched population with aggressive drainage and active exclusion of trapped lung prior to talc, the overall pleurodesis rate at day 70 was still only 50% compared to − 5–80% pleurodesis success in conventional slurry or poudrage (reference TAPPS). Nonetheless, IPC-PLUS provides a useful therapeutic option for individuals preferring an ambulatory pathway who might also be motivated to have their IPC removed early. Of note, there was no significant increase in IPC blockage rates or adverse events in the talc group, including no increased development of loculated effusions.

Current ongoing trials on the horizon include the AMPLE-3 study being conducted by the Australia New Zealand group which is the first randomised trial to compare IPC (+/- talc) versus VATS pleurodesis in those who are fit for surgery with a primary outcome of the need for further ipsilateral pleural interventions over 12 months [76]. TACTIC trial is an unblinded multicentre RCT carrying out in United Kingdom, compares the combination use of thoracoscopic talc poudrage (TTP) with an IPC versus TTP alone with co-primary outcomes of time spent in hospital and mean breathlessness score over 4 weeks post-procedure.

### Fibrinolysis in MPE

Not all MPEs appear to be free and a subset of MPEs that have septations are indicative of advanced disease that represent a higher mortality [86]. The TIME 3 trial explored the role of breaking down these septations with urokinase followed administration of talc, being the time to pleurodesis failure alongside change in dyspnoea scales the primary outcomes. This study showed that there was no difference in the primary outcomes between the urokinase group versus placebo despite evidenced radiological improvements in the former [86]. Based on the results of this trial it was suggested, for loculated MPEs group, ambulatory management based on both serial pleural aspirations or IPCs, is considered the first line of management due to a shorter life expectancy and aiming to avoid hospital admission [86]. It is likely that further study is needed to fully understand the role of fibrinolytics in MPE but current evidence would suggest that alternative palliative measures should be used in these patients. However, in highly selected patients with septated MPE and significant symptom burden, a trial of intrapleural fibrinolytics is reasonable to try to alleviate breathlessness. This is also a reasonable approach in the context of patients with a septated MPE and an IPC in situ if attempts at unblocking the catheter with a heparin saline flush do not improve drainage [4].

**Fig. 2:**
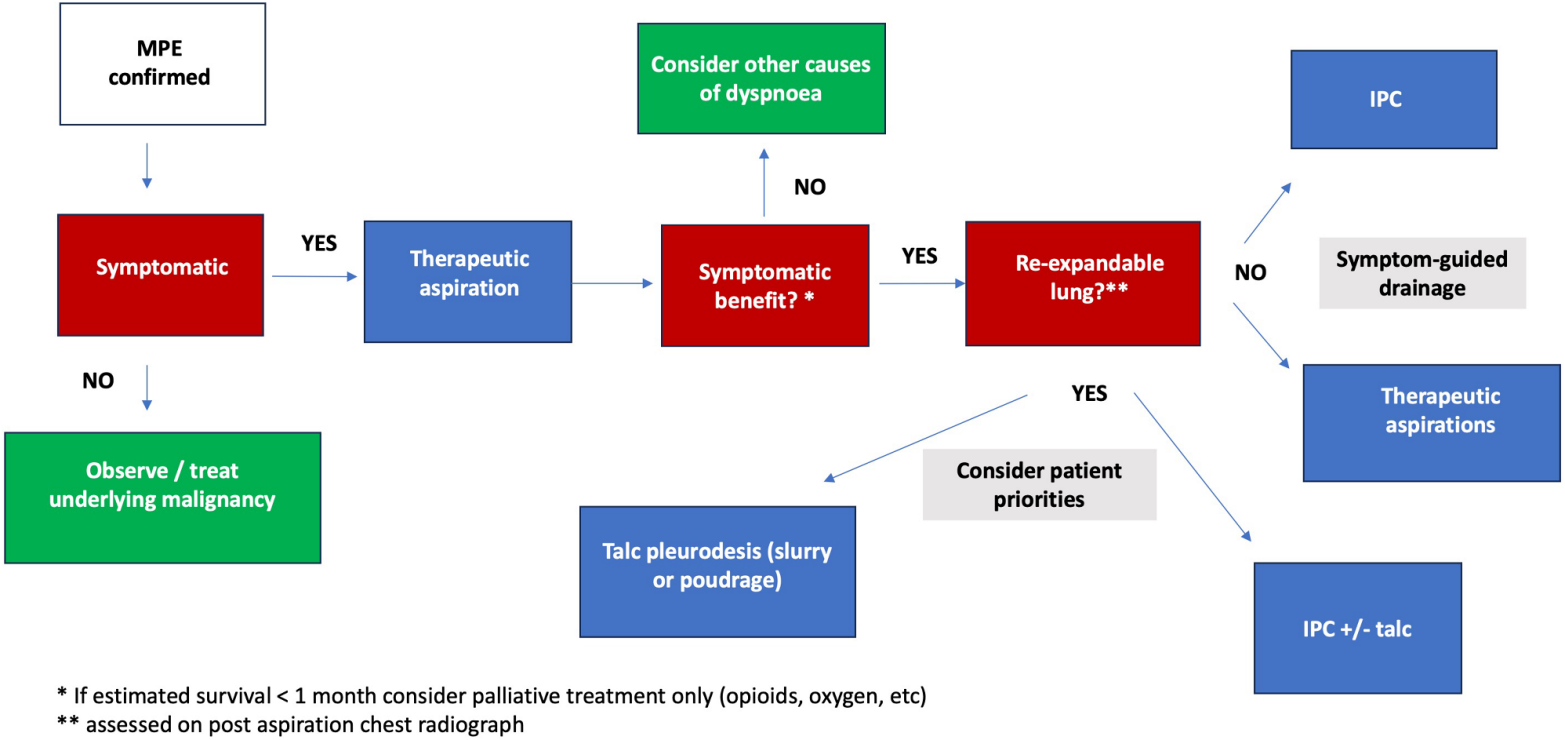
Management approach in MPE

In summary, management of MPEs is not straightforward and we believe that a patient centred approach should be adapted according to patients and care givers needs and open discussions over the management options should be encouraged (Fig. 2). Patient leaflets and online resources such as mypleuraleffusionjourney.com should be utilized so as to facilitate an informed decision [83].

## Prognostication in MPE

There are now a variety of management options available, as highlighted above, for managing MPEs however each intervention has its own implications in terms of burden on the patient [87] and burden of cost on the healthcare system [88]. Being able to select the appropriate patients that are eligible for intervention can prove to be quite challenging as going off of clinical picture and ECOG scores alone can be misleading [8]. Prognostication scores can be used when explaining prognosis to patients and families and be used to guide management of MPEs [8].

The LENT score was the first externally validated score for MPE, it has a score range of 0–7 which can be subdivided into low risk (score 0–1), moderate risk (score 2–4) and high risk (5–7). The LENT score consists of 4 parameters: pleural fluid lactate dehydrogenase (LDH) levels, Eastern Cooperative Oncology Group (ECOG) performance score, serum neutrophil- lymphocyte ratio, and tumour type. The risk stratification score can be used to estimate the 1-, 3- and 6-months mortality. High levels of pleural fluid LDH and high level of serum neutrophil-lymphocyte ratio conferred a higher score, cancer types such as lung and breast scored higher score of 2 and 1 respectively indicating a more aggressive disease process [8]. One of the main limitations of this score is that it only groups individuals into three categories, it also does not differentiate between the different subsets of tumour types based on cell markers [88].

The PROMISE (pleurodesis response markers in malignant pleural effusion) score was the second to be externally validated for prognostication of MPEs which combined the use of clinical, radiological and biological markers and all the parameters were independently associated with survival [9]. Although cancer type and ECOG PS are included, like the LENT scoring system, it adds further parameters such as use of chemotherapy or radiotherapy in managing the tumour, parameters that are markers of inflammation like white cell count and C- reactive protein and measured haemoglobin levels. There are 2 variations of this score PROMISE: clinical (which has the above parameters) and PROMISE biological which includes pleural fluid TIMP-1 (tissue inhibitor of metalloproteinase 1) levels. Based on the scores, patients are allocated into range of categories from A to D and 3-month mortality predicted. Category A has a less than 25% risk, followed by 25–50% for category B, 50–75% and > 75% for categories C and D respectively.

These tools provide a useful armoury to clinicians where the information around survival estimates is valuable in discussions with patients or in planning treatments. However, the impact of these scores on clinical-decision making is yet to be evaluated and neither have been assessed in their ability to improve patient outcomes. The priority remains to ensure all patients with MPE are approached in a multidisciplinary way including early involvement of specialist palliative care services [4].

## Future directions

Great progress has been made in building what is now a robust evidence base to inform a number of management strategies in MPE but nonetheless these still primarily focus on optimal drainage strategies. Over the next decade, more upstream research is needed to advance our understanding of the mechanistic drivers of MPE formation on a cellular level. Advances in immunological and intrapleural therapies pose exciting therapeutic directions that may offer targeted pharmacological treatment of MPE and it remains to be seen what effect the increasing use of immune checkpoint inhibitors and targeted therapies will have on the incidence of MPE. Early translational work has suggested that pleural fluid may not be an innocent bystander and may have pro-growth properties promoting cancer cell proliferation thus requiring pro-active drainage beyond palliative symptom control [15]. As we increasingly define treatment pathways based on patient preference, there is a need for high quality qualitative data exploring the patient experience along the varied treatment strategies to inform discussions and to empower patients to make informed decisions.

## Conclusion

In conclusion, we have learned a great deal about the mechanisms and optimal management of malignant pleural effusion via research over the last 20 years. However, much remains unknown and further research is indicated across a number of areas, to better understand underlying biology of pleural fluid production and, thus, to achieve more targeted therapies.

Diagnosis of MPE has significantly advanced and will do further with more sensitive molecular techniques including the so called “liquid biopsy”. However, as pleural fluid cytology alone is poorly sensitive, and even when positive is often insufficient to provide full enough oncological information for treatment, nowadays biopsy remains a key investigation in the work up of potential MPE.

Our understanding of optimal treatment intervention in MPE has been greatly advanced by recent high quality randomised trials and there are a number of potential options. In future years, it is likely that combined diagnostic and therapeutic procedures allowing near total outpatient management of MPE will become popular – but such innovations require high quality evidence to assess their patient focused and diagnostic benefits and risks.

## Data Availability

No datasets were generated or analysed during the current study.

## Abbreviations

ALK: Anaplastic lymphoma kinase
ANG-1 and 2: Angiopoietin 1 and 2
BRAF: v-raf murine sarcoma viral oncogene homolog B
CCL: Chemokine (c-c motif) ligand
CT: Computed tomography
EGFR: Epidermal growth factor receptor
EML4/ALK: Echinoderm microtubule-associated protein-like 4 gene/anaplastic lymphoma kinase gene
IPC: Indwelling pleural catheter
ITT: Intention to Treat
KRAS: Kirsten rat sarcoma virus
LAT: Local anaesthetic thoracoscopy
MMP: Matrix metalloproteinases
MPE: Malignant pleural effusion
MT: Medical thoracoscopy
NEL: Non-Expansile lung
NF-κB: Nuclear factor kappa-light-chain-enhancer of activated B cells OPN:osteopontin
PET-CT: 18 F-fluorodeoxyglucose – positron emission tomography
STAT: Signal transducer and activator of transcription
TNF: Tumor necrosis factor
TTP: Thoracoscopic talc poudrage
TUS: Thoracic ultrasound
VEGF: Vascular endothelial growth factor
VATS: Video-Assisted thoracoscopic surgery
